# *BRAF*-mutant microsatellite-stable rectal cancer with acquired *KRAS* mutation leading to drug resistance in liver metastasis

**DOI:** 10.1007/s13691-024-00678-2

**Published:** 2024-04-18

**Authors:** Kunitoshi Shigeyasu, Hideki Yamamoto, Toshiaki Takahashi, Kazuya Moriwake, Masashi Kayano, Sho Takeda, Yuki Matsumi, Yuzo Umeda, Yoshitaka Kondo, Fuminori Teraishi, Kazuya Yasui, Tomokazu Fuji, Shunsuke Kagawa, Toshiyoshi Fujiwara

**Affiliations:** 1 Department of Gastroenterological Surgery, Okayama University Graduate School of Medicine, Dentistry, and Pharmaceutical Sciences, Okayama, Japan; 2 Department of Clinical Genomic Medicine, Okayama University Graduate School of Medicine, Dentistry, and Pharmaceutical Sciences, Okayama, Japan

**Keywords:** *BRAF*, Colorectal cancer, *KRAS*, Resistance

## Abstract

*BRAF*-mutant microsatellite-stable colorectal cancer (CRC), metastasized to distant sites, is associated with a poor prognosis. However, the BEACON CRC regimen, comprising a BRAF inhibitor, MEK inhibitor, and anti-EGFR antibody, offered a prolonged prognosis. Nonetheless, resistance to this regimen may occur, as observed in our reported case of CRC, where a *KRAS* mutation was identified in addition to the *BRAF* V600E mutation. Here, we present a case of 74-year-old woman with rectal cancer (pT4bN1bM0 Stage IIIc) harboring the *BRAF* V600E mutation. After resection of the primary tumor and during adjuvant chemotherapy using CAPOX (capecitabine and oxaliplatin), liver and lung metastases became apparent, and a companion diagnosis test revealed the presence of a *BRAF* V600E mutation. The new lesions were deemed resistant to the CAPOX regimen, and we decided to introduce encorafenib and cetuximab. After resection of liver metastases, encorafenib and cetuximab were reintroduced, but a new lesion appeared in hepatic S7, indicating resistance to the encorafenib and cetuximab regimen. The resistant liver metastasis was subsequently resected. To elucidate the resistance mechanism, we conducted a comprehensive analysis using the FoundationOne CDx cancer gene panel test, revealing the presence of a *KRAS* Q61H mutation alongside the *BRAF* V600E mutation. Subsequent liquid biopsy after liver recurrence confirmed the persistence of the *KRAS* Q61H mutation. Our results highlight the significance of cancer genome profiling tests (CGP tests) and liquid biopsies in guiding treatment strategies for *BRAF*-mutant colorectal cancer. Therefore, CGP testing offers valuable information for treatment, even if it does not lead to new drug administrations.

## Introduction

*BRAF*-mutant microsatellite-stable colorectal cancer (CRC) with distant metastasis typically carries a poor prognosis [1, 2]. However, a more favorable prognosis can be expected with the advent of the BEACON CRC regimen, combining a BRAF inhibitor, MEK inhibitor, and anti-EGFR antibody [3].

Concurrently, the cancer genome profiling test (CGP test) analyzes the cancer genome in tumors to identify genetic mutations contributing to cancer initiation and growth, aiming to select drugs and treatments tailored to these mutations. Covered by insurance in Japan, the CGP test is now widely used for colorectal cancer treatment. According to the Ministry of Health, Labour, and Welfare’s FY2020 performance survey, only 8.1% of patients achieved therapy matching their genetic abnormality after CGP testing [4]. The identification rate of patients with target genomic abnormalities through CGP testing is not high, and there are limited clinical trials involving pediatric patients. Consequently, the reach of these drugs is restricted. Ongoing challenges include the development of new CGP tests and the expansion of drug indications.

Nevertheless, the practical application of this information in daily medical practice remains unclear, particularly when data do not lead to the discovery of new drugs or clinical trials. To address this challenge, we have developed a strategy that combines conventional companion diagnostics with CGP testing to enhance drug selection for colorectal cancer chemotherapy. In this study, we present a case of colorectal cancer that demonstrated resistance to the encorafenib and cetuximab regimen, attributed to the presence of a *KRAS* mutation in addition to *BRAF*. The information obtained from both companion diagnosis and CGP testing proved beneficial for selecting the appropriate drug in the later stages of treatment. Through this case study, we aim to elucidate and demonstrate the utility of the CGP test in improving colorectal cancer treatment strategies.

## Case report

**Fig. 1:**
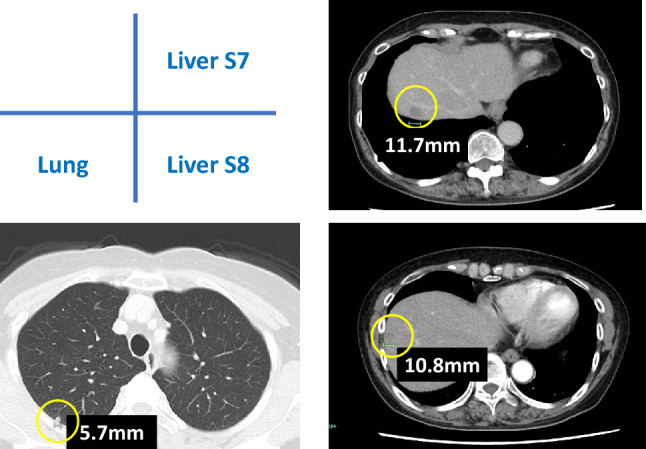
Computed tomography image before initial liver resection

A 74-year-old woman diagnosed with rectal cancer (pT4bN1bM0 Stage IIIc) underwent resection of the primary tumor, followed by postoperative adjuvant chemotherapy using CAPOX (capecitabine and oxaliplatin). During adjuvant chemotherapy, liver and lung metastases became apparent, and a companion diagnosis test (RASKET-B) revealed the presence of a *BRAF* V600E mutation. The new lesions were deemed resistant to the CAPOX regimen, and we decided to introduce encorafenib and cetuximab. Notably, tumor markers decreased, and metastases diminished after 4 months of chemotherapy (Fig. 1). The drug was well-tolerated, and no drug reduction or withdrawal was necessary.

**Fig. 2:**
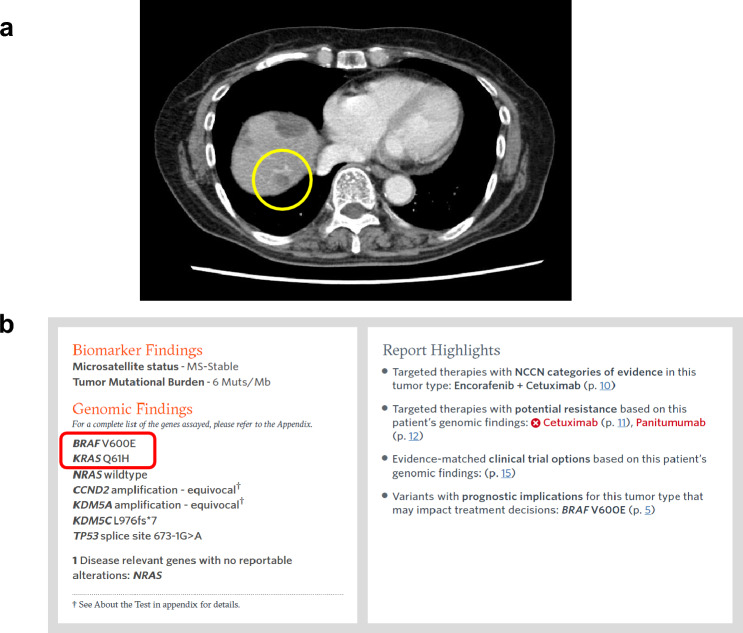
Second resection of liver metastases. **a** Emergence and growth of a new metastatic lesion in liver S7. **b** Detection of *KRAS* Q61H in addition to *BRAF* V600E in the new metastases of liver S7

To achieve complete resection of distant metastases, the patient underwent partial hepatic resection involving segments S3, S5, S6, and S7. The postoperative course was favorable, and the patient recovered within 3 weeks. Despite this, as lung metastases persisted, postoperative combination therapy with a encorafenib and cetuximab was reintroduced. The patient was scheduled for resection of lung metastases if no new lesions appeared. While the lung metastases remained in remission, a subsequent liver recurrence was observed 1 month after the resection of liver metastasis in segment S7 (Fig. 2a). Notably, only this site showed a rapid growth trend, in contrast to the stable lung metastasis. Although the cause was unknown, the liver metastasis was deemed resistant to the encorafenib and cetuximab, prompting its surgical resection.

The mechanism behind the specific resistance of the remaining liver metastases to the encorafenib and cetuximab needs clarification before devising subsequent chemotherapy. To clarify the reason for drug resistance, we analyzed the resected liver metastases using the FoundationOne CDx cancer gene panel test, using the second set of resected liver metastases. Interestingly, in addition to the *BRAF* V600E mutation (Variant Allele Frequency, VAF: 60.7%), a *KRAS* Q61H mutation (VAF: 28.6%) was identified (Fig. 2b). This *KRAS* mutation had not been initially identified using the RASKET-B test during the primary resection. Therefore, it was considered to be an acquired mutation arising from the development of a new liver metastasis. Previous reports indicated that *KRAS* Q61H is associated with inducing resistance to anti-EGFR antibodies [5]. This acquired mutation may play a pivotal role in the observed resistance to the encorafenib and cetuximab, which includes anti-EGFR antibodies.

The resection of liver metastasis caused the complete elimination of cancerous tissue with *KRAS* Q61H from the patient’s body. To confirm that the *RAS* was wild-type, OncoBEAM RAS CRC was employed as a liquid biopsy. This test confirmed the absence of residual colorectal cancer cells resistant to the encorafenib and cetuximab with *KRAS* Q61H. However, considering the high risk of recurrence in *BRAF*-mutant cancer, the patient continued on a combination therapy of encorafenib and cetuximab. After 3 months, another OncoBEAM RAS CRC liquid biopsy was performed, revealing the persistence of the *KRAS* Q61H mutation. A subsequent computed tomography unveiled residual liver recurrence and peritoneal dissemination. The patient was depressed and mentally unstable; therefore, we declined to administer the irinotecan-containing regimen. In all, 2 months of regorafenib maintained her in stable disease.

## Discussion

The BEACON CRC trial was an international, multicenter, randomized, open-label, phase III clinical trial that enrolled patients with *BRAF* V600E mutation-positive CRC who had progressed after one or two prior therapies. Participants received either the triple combination of encorafenib, binimetinib, and cetuximab, the double combination of encorafenib and cetuximab, or standard treatments (control group) such as irinotecan with cetuximab or FOLFIRI (fluorouracil and irinotecan) with cetuximab [1, 2].

The primary endpoints of the trial were the objective response rate and overall survival (OS) in the three-drug combination arm compared to the control arm. The secondary endpoint was OS in the two-drug combination arm compared to the control arm. Moreover, 665 patients were randomized from May 2017 to January 2019. At an interim analysis with a data cutoff date of February 11, 2019 (median observation time 7.8 months), the OS in the three-drug combination arm was significantly better than that in the control arm (median OS 9.0 months vs. 5.4 months, hazard ratio (HR) 0.52, 95% confidence interval (CI) 0.39–0.70, *P* < 0.001). The two-drug combination arm also showed significant improvement compared to the control arm (median OS 8.4 vs. 5.4 months, HR 0.60, 95% CI 0.45–0.79, *P* < 0.001).

Therefore, both the three-drug combination (encorafenib, binimetinib, and cetuximab) for *BRAF* V600E mutation-positive colorectal cancer and the two-drug combination (encorafenib and cetuximab) are considered standard treatment options for *BRAF* V600E mutation-positive colorectal cancer. As a result, they are available for second-line treatment or later in Japan.

The evaluation method for resistance when using the BEACON CRC regimen has not yet been clearly defined. Previous studies have shown that acquired resistance to BRAF-directed therapy in *BRAF* V600E colorectal cancer patients is often caused by genomic alterations (e.g., *RAS* mutations) that lead to reactivation of MAPK signaling [6–9]. Corcoran et al. demonstrated that almost half of patients showed emergence of *KRAS* or *NRAS* mutations in cell-free DNA at the time of disease progression in a larger cohort of *BRAF* V600E CRC patients [9]. There is also a need for further clarification of the resistance mechanism to BEACON CRC.

In this case, we submitted metastases rather than the originating tumors for oncogene panel testing. This decision allowed us to elucidate the resistance mechanism triggered by the acquisition of *KRAS* mutations, providing crucial information essential for selecting the appropriate treatment regimen. The practice of screening for *RAS* mutations using liquid biopsy during the administration of the BEACON CRC regimen offers a more accessible means of predicting resistance [5]. *RAS* mutation screening by liquid biopsy may be a more convenient method of predicting resistance to the BEACON CRC regimen.

The BEACON CRC regimen is approved in Japan for second-line treatment or later. While FOLFOXIRI (fluorouracil, oxaliplatin, and irinotecan) and bevacizumab regimen has been reported as more effective for first-line treatment [10], a recent meta-analysis showed that Doublet might be sufficient for first-line treatment [11]. CAPOX plus bevacizumab and mFOLFOX-6 (fluorouracil and oxaliplatin) plus bevacizumab are among the most common regimens for first-line treatment. In cases where patients develop resistance to the BEACON CRC regimen, alternative options include trifluridine/tipiracil, regorafenib, and repeat administration of irinotecan. Due to the rapid progression observed upon resistance, a switch to other regimens is crucial when progression occurs.

The question arises regarding the potential rechallenge with the BEACON CRC regimen. We need to wait for the results of the TRIDENTE study (jRCTs031210511). This is a phase II trial exploring the efficacy and safety of the combination therapy rechallenge of encorafenib plus binimetinib plus cetuximab in patients with *BRAF* V600E-mutant unresectable advanced or recurrent colorectal cancer who have previously been treated with combination therapy including encorafenib and cetuximab. Additionally, the efficacy of cetuximab, encorafenib, and binimetinib after failure of adjuvant therapy remains unknown. Prospective studies are ongoing, and results are awaited [12].

Given the nature of this study as a case report, generating evidence is challenging. Nonetheless, our findings indicate that CGP testing and liquid biopsy in *BRAF*-mutant cancer may offer valuable insights into determining treatment strategies. Even if not leading to the introduction of new drugs, CGP testing may provide useful information for optimizing treatment approaches.

## Data Availability

All data generated or analyzed during this study are included in the published article.
